# Investigating Environmental Links Between Parent Depression and Child Depressive/Anxiety Symptoms Using an Assisted Conception Design

**DOI:** 10.1016/j.jaac.2011.01.015

**Published:** 2011-05

**Authors:** Gemma Lewis, Frances Rice, Gordon T. Harold, Stephan Collishaw, Anita Thapar

**Affiliations:** a Child and Adolescent Psychiatry Section and the Medical Research Council Centre for Neuropsychiatric Genetics and Genomics, Cardiff University; b Division of Psychology and Language Sciences, University College London; c College of Biological Sciences, University of Leicester

**Keywords:** depression, anxiety, transmission, environmental, genetic

## Abstract

**Objective:**

Links between maternal and offspring depression symptoms could arise from inherited factors, direct environmental exposure, or shared adversity. A novel genetically sensitive design was used to test the extent of environmental links between maternal depression symptoms and child depression/anxiety symptoms, accounting for inherited effects, shared adversity, and child age and gender.

**Method:**

Eight hundred fifty-two families with a child born by assisted conception provided questionnaire data. Mothers and fathers were genetically related or unrelated to the child depending on conception method. Parental depression symptoms were assessed using the Hospital Anxiety and Depression Scale. Child depression/anxiety symptoms were assessed using the Short Mood and Feelings questionnaire and six items tapping generalized anxiety disorder symptoms. Associations between maternal and child symptoms were examined separately for genetically unrelated and related mother–child pairs, adjusting for three measurements of shared adversity: negative life events, family income, and socioeconomic status. Analyses were then run separately for boys and girls and for children and adolescents, and the role of paternal depression symptoms was also examined.

**Results:**

Significant associations between parent and child symptoms were found for genetically unrelated mother–child (*r* = 0.32, *p* < .001) and father–child (*r* = 0.17, *p* < .05) pairs and genetically related mother–child (*r* = 0.31, *p* < .001) and father–child (*r* = 0.23, *p* < .001) pairs and were not explained by the shared adversity measurements. Environmental links were present for children and adolescents and were stronger for girls.

**Conclusions:**

The transmission of depression symptoms is due in part to environmental processes independent of inherited effects and is not accounted for by shared adversity measurements. Girls may be more sensitive to the negative effects of maternal depression symptoms than boys through environmental processes.

Children of depressed parents are two to three times more likely than the offspring of nondepressed controls to exhibit elevated levels of depression and anxiety symptoms and depressive disorder.^1^ Despite consistent evidence that depression is familial, few studies have tested the processes underlying intergenerational transmission of symptoms by teasing apart competing explanations. First, exposure to parent depression symptoms may have a direct environmental effect on children. Second, links between parent and child symptoms could arise through inherited factors. Third, shared exposure to adversities such as bereavement, divorce, or poverty may increase risk for depression in parents and children, accounting for observed transmission effects. A greater understanding of the pathways involved in intergenerational transmission can help in the identification of modifiable risk factors for child/adolescent depression symptoms that could be targeted in preventive and therapeutic interventions.

Few studies have used genetically sensitive designs to estimate the influence of inherited factors and separate environmental from genetic effects.^2-5^ One study did not assess maternal depression directly^5^ and another reported inconsistent findings depending on the informant used.^4^ Two studies have directly tested environmental transmission of maternal depression.^2,3^ Tully et al.^2^ reported significant associations between maternal major depression and child major depression in adopted and nonadopted families, with greater associations in nonadopted mother–child pairs, suggesting environmental and genetic effects. Using a children-of-twins design, Silberg et al.^3^ found that the intergenerational transmission of maternal depression symptoms was due to family environmental factors but reported no genetic influence. Findings have suggested that parent depression symptoms may exert environmental risk effects on child depression symptoms. However, previous studies have not ruled out the possibility that specific measurements of shared environmental adversity (affecting parents and offspring) contribute to this environmental link or account for the association between maternal depression and child symptoms.

Further research on the mechanisms underlying intergenerational transmission of depression is required. In addition to the role of shared adversity, such research should consider the effects of child gender and age, which have not been examined using genetically sensitive designs. There is evidence that different factors may be involved in the etiology and intergenerational transmission of depression for boys and girls.^6^ Some studies have found that the association between maternal and offspring depression is stronger in daughters than in sons.^7,8^ However, other studies have not reported significant gender differences.^9^ The etiology of child and adolescent depression may also vary according to the developmental stage of children. A consistent finding from twin research has been that genetic influences on depression symptoms are greater in adolescents, whereas shared environment is more influential in children.^10^ This suggests that noninherited factors could play a more prominent role in the intergenerational transmission of depression symptoms for younger children. There is also consistent evidence that anxiety and depression symptoms have shared genetic liability and that, in prepubertal children, this liability more commonly manifests as anxiety.^11^ This suggests the need to examine anxiety and depression in the offspring of depressed parents.

The present study, based on a sample of children conceived by assisted reproduction, aimed to test whether there are environmentally mediated links between parental depression and child depression/anxiety, and whether they are accounted for by exposure to three measurements of shared adversity-negative life events, family income, and socioeconomic status. In addition, the effects of child age and gender were investigated. The sample allows tests of environmental and genetic transmission using principles akin to the classic behavioral genetic adoption design: comparisons of associations between genetically unrelated and related parent–child pairs. Associations of parent and child symptoms in genetically unrelated dyads indicates the presence of environmental transmission, implicating the role of the shared environment (C), whereas evidence for genetic effects would be shown if associations are greater in related versus unrelated pairs (G). A recent study using this sample examined depression and conduct symptoms in children and found preliminary evidence of environmental links.^12^ However, this study focused on mediation of the association by parent–child warmth and hostility and did not rule out the possibility that alternative influences such as shared adversity may account for effects or that associations may differ according to child age and gender. It was hypothesized in the present study that maternal depression symptoms, in addition to showing inherited transmission, would have direct environmental links not accounted for by shared adversity, with symptoms of depression and anxiety in children, and that the environmental association would be stronger in girls and preadolescent children younger than 11 years.

## Method

### Sample

The sample consisted of families recruited from 19 fertility clinics across the United Kingdom and one in the United States who had undergone in vitro fertilization (IVF), with children born from 1994 through 2002. Twenty-two clinics were approached and 19 agreed to participate, representing a clinic response rate of 86%. Children included in this report were conceived by one of four IVF methods: homologous (genetically related to both parents), sperm donation (genetically related to mother only), egg donation (genetically related to father only), or embryo donation (genetically related to neither parent). All gamete donors were unrelated to parents. One child from each family participated. For multiple births, parents were asked to report on the firstborn child. Data were collected by postal questionnaire from mothers and fathers on two occasions (Time 1 [T1], 2006; Time 2 [T2], 2009). Questionnaires included a range of measurements assessing details of the pregnancy, child psychopathology, family environment, and parental psychopathology.

One thousand one hundred fifty-eight families were sent questionnaires at T1. Responses were received from 852 families (74% response rate). Seven hundred eighty-one families agreed to be contacted about further studies and these families were mailed follow-up questionnaires at T2. At T2, responses were received from 482 families (62% response rate). Participants who dropped out at T2 did not differ from those who remained in the study in maternal depression symptoms at T1 (mean retained in study = 4.24, standard deviation [SD] = 3.04; mean dropped out of study = 4.46, SD = 3.10, *t*_848_ = 1.04, *p* = .30) or child depression/anxiety as rated by the mother (mean retained in study = 4.68, SD = 3.96; mean dropped out of study = 4.50, SD = 4.57, *t*_847_ = 0.61, *p* = .55) and by the father (mean retained in study = 4.34, SD = 4.29; mean dropped out of study = 3.93, SD = 4.29, *t*_568_ = 1.15, *p* = .25). Attrition was not associated with genetic relatedness to the child (χ^2^_1_ = 0.94, *p* = .33). The effects of potential biases caused by sample attrition were investigated by running T1 analyses on the subgroup retained across waves; the same pattern of results was found (available on request).

Comparisons with U.K. national norms have shown that this sample does not differ from the general population in psychological adjustment, with minimal differences found between the conception groups.^13^ The present study used data from families in which the mother had responded (n = 852, 431 boys, 421 girls at T1; n = 459, 231 boys, 228 girls at T2). At T1 the numbers in each conception group were 436 homologous, 208 sperm donation, 173 egg donation, and 35 embryo donation, and at T2 the numbers were 254 homologous, 99 sperm donation, 91 egg donation, and 15 embryo donation. The genetically unrelated group consisted of 208 mother–child pairs (egg, embryo donation) and the genetically related group consisted of 644 mother–child pairs (homologous, sperm donation). Children were 4 to 10 years old (mean = 6.3 years) at T1 and 7 to 13 years old (mean = 9.9 years) at T2. Mothers were 27 to 61 years old (mean = 41.44 years) and fathers were 28 to 76 years old (mean = 44.3 years) at T1. Most children (91%) resided with both parents.

### Measurements

#### Maternal Depression Symptoms

Maternal depression symptoms were assessed using the seven-item self-report depression subscale of the Hospital Anxiety and Depression Scale.^14^ This subscale showed good internal consistency in this sample (α = 0.75 at T1 and α = 0.70 at T2) and is well validated.^15^ Scores on the subscale range from 0 through 21, with higher scores representing greater depression symptoms. A recommended clinical cutpoint higher than 8 has been validated in clinical and community samples.^15^

#### Child Anxiety and Depression Symptoms

Child depression symptoms were assessed using parent ratings (mothers and fathers separately) on the Short Mood and Feelings Questionnaire (SMFQ). Child self-reports were not obtained because of the children's young age and for ethical reasons (children might not be aware of parental IVF treatment). The SMFQ is a 13-item self-report measurement of the presence and severity of *DSM-IV* depression symptoms, which showed good internal consistency in this sample (α = 0.73 for mothers and 0.79 for fathers at T1 and α = 0.75 for mothers at T2), and is a well-validated measurement in clinical and community samples.^16^ Total scores range from 0 through 26, with higher scores representing greater depression symptoms and the recommended clinical cutpoint is higher than 8.^16^ Child anxiety symptoms were assessed using parent reports on six *DSM-IV* items for generalized anxiety disorder over the previous 3 months (worries, restless, easily tired, irritable, difficulty concentrating, problems sleeping). The anxiety scale showed good internal consistency in this sample (α = 0.73 for mothers and 0.67 for fathers at T1 and α = 0.76 for mothers at T2). Anxiety scores ranged from 0 through 12, with higher scores representing greater anxiety symptoms. A combined depression/anxiety scale was created by summing SMFQ and *DSM-IV* anxiety items and showed good internal consistency (α = 0.83 for mothers and 0.85 for fathers at T1 and α = 0.87 for mothers at T2). Scores on the composite measurement ranged from 0 through 38, with higher scores representing more depression/anxiety symptoms.

#### Negative Life Events

A 35-item Life Events Checklist^17^ completed by mothers measured the occurrence of a range of significant events in the child's life during the previous year such as “death of a parent, grandparent or sibling.” Fourteen independent negative life events likely to affect mother and child were used.

#### Family Income

Mothers and fathers were each asked to rate the approximate gross family income on a six-point Likert scale ranging from <10,000 to >60,000 a year.

#### Family Socio-occupational Class

Families were classified according to the occupation of the main earner (Office for National Statistics, 2000).

### Statistical Analyses

The sample was divided into two groups: genetically unrelated mother–child pairs (egg and embryo donation) and genetically related mother–child pairs (homologous IVF and sperm donation). Association between maternal depression symptoms and child depression/anxiety symptoms was calculated separately in each group. The principle of the IVF design is consistent with the classic behavioral genetic adoption design: The presence of an association in unrelated mother–child pairs would provide evidence of environmental transmission because inherited links are removed, therefore indicating the role of a shared environment (C). If an association is seen only in related mother–child pairs, then this indicates inherited transmission suggesting that parent–child links are due to genetic factors (G). When an association is seen in both groups but is stronger in the related group, this indicates genetic and environmental links (G + C). Associations were calculated using the Pearson product-moment correlation coefficient (*r*) since variables were continuous and normally distributed after transformation. The difference in the magnitude of correlation coefficients across groups was tested using the Fisher *R* to *Z* transformation. This test converts Pearson *r* to the normally distributed variable *Z*, enabling a comparison of the strength of associations. Associations were then retested in each group controlling for differences in maternal age, child age, and family structure (two-parent or single-parent family).

To test whether associations differed according to child age, analyses were conducted separately at T1 (mean child age = 6.3 years) and T2 (mean child age = 9.9 years). The sample was then split at T2 into children (≤11 years old) and adolescents (>11 years old). All analyses were conducted using mother and father ratings of child symptoms to control for shared rater effects; however, father ratings were available only at T1 because of low paternal response at T2. Associations between maternal depression symptoms and child depression/anxiety symptoms (mother and father rated) were then tested adjusting for three measurements of shared adversity: independent stressful life events, family income, and family social occupational class. The role of shared adversity was examined at T1 because of the larger available sample.

The role of child gender was tested next. Mother–child associations were examined separately for boys and girls at both time points (using mother and father ratings at T1 and mother ratings only at T2) to test whether the effects of gender varied by child age.

The role of paternal depression symptoms was next examined. First, associations between maternal depression symptoms and child depression/anxiety were tested controlling for paternal depression symptoms to rule out the possibility that father psychopathology could have been driving mother–child associations. Second, the association between paternal depression symptoms and child depression/anxiety was tested separately in genetically related (homologous IVF, egg donation) and unrelated (sperm and embryo donation) father–child pairs controlling for shared adversity.

## Results

### Demographic Characteristics and Parent and Child Symptoms

Children were significantly younger in the mother unrelated group (egg and embryo donation; mean age T1 unrelated group = 6.00 years; mean age T1 related group = 6.35 years, *t*_850_ = 3.5, *p* < .001). A larger percentage of children lived with their mothers only in the unrelated (10%) versus related (5%) group and this difference was statistically significant (χ^2^_1_ = 5.1, *p* < .05). Mothers in the unrelated group were significantly older; mean maternal age in the unrelated group was 44.91 years and in the related group, 40.31 years (*t*_849_ = 12.79, *p* < .001). There were more multiple births in the unrelated versus the related group (24% versus 21%) but this difference was not statistically significant (χ^2^_1_ = 0.76, *p* > .05).

Mean maternal depression symptoms did not differ according to relatedness at T1 (mean unrelated = 4.63, SD = 3.06, range = 0–15, n = 208; mean related = 4.24, SD = 3.06, range = 0–18, n = 642; *t*_848_ = 1.59, *p* = .11) or T2 (mean unrelated = 4.58, SD = 3.49, range = 0–18, n = 106; mean related = 4.04, SD = 3.19, range = 0–21, n = 353; *t*_457_ = 1.48, *p* = .14). Mean paternal depression symptoms did not differ at T1 (mean unrelated = 3.86, SD = 2.70, range = 0–14, n = 167; mean related = 3.87, SD = 3.12, range = 0–21, n = 410; *t*_575_ = 0.4, *p* = .97). Mothers reported significantly higher mean depression symptoms than fathers (*t*_576_ = 5.64, *p* < .01). Parent depression symptoms were positively skewed so were square root transformed. Child depression/anxiety symptoms also showed no significant differences across groups according to mother ratings (mean unrelated = 4.04, SD = 3.54, range = 0–19, n = 208; mean related = 4.78, SD = 4.44. range = 0–19, n = 641; *t*_847_ = 2.18, *p* = .06, at T1; mean unrelated = 5.18, SD = 4.21, range = 0–21, n = 105; mean related = 4.91, SD = 4.82, range = 0–30, n = 352; *t*_455_ = 0.522, *p* = .60, at T2) and father ratings (mean unrelated = 3.66, SD = 3.79, range = 0–20, n = 133; mean related = 4.32, SD = 4.37, range = 0–32, n = 437; *t*_568_ = 1.58, *p* = .12). Fifteen percent of mothers and 9% of fathers scored above the clinical cutpoint on the Hospital Anxiety and Depression Scale at T1 and 14% of mothers at T2. Five percent of children scored above the clinical cutpoint on the SMFQ at T1 according to mother and father ratings and 7% at T2 according to mother ratings.

#### Testing for Inherited and Environmental Links Between Maternal Depression Symptoms and Child Depression/Anxiety

The association between maternal depression symptoms and child depression/anxiety was significant in both groups for mother-rated (unrelated: n = 207, *r* = 0.32, *p* < .001; related: n = 639, *r* = 0.31, *p* < .001) and father-rated (unrelated: n = 132, r = 0.26, *p* < .01; related: n = 436, *r* = 0.22, *p* < .001) child symptoms at T1. Correlation coefficients did not differ across related and unrelated groups according to Fisher *R* to *Z* for mother ratings (*Z* = 0.28, *p* = .39) or father ratings (*Z* = 0.31, *p* = .38) of child symptoms. Correlation coefficients did not differ across raters in unrelated (*Z* = 0.66, *p* = .25) and related (*Z* = 1.56, *p* = .06) groups. To control for known group differences, maternal age, child age, and family structure were then entered as covariates at T1. Associations remained significant for mother (unrelated: n = 207, *r* = 0.33, *p* < .001; related: n = 638, *r* = 0.31, *p* < .001) and father (unrelated: n = 132, *r* = 0.26, *p* < .01; related: n = 436, *r* = 0.22, *p* < .001) ratings of child symptoms. The pattern of results was the same when child depression and anxiety symptoms were examined separately (Table S1, available online).

#### Testing Contribution of Shared Adversity

The association at T1 was examined controlling for negative life events likely to affect mother and child, family income, and family social occupational class (Table 1). Associations remained significant and unattenuated after controlling for each adversity separately across groups for mother- and father-rated child symptoms. When all three variables were entered into a multiple partial correlation analysis with maternal depression symptoms, associations for mother- and father-rated child symptoms remained significant in the two groups.

#### Age Effects

The analyses at T2 when children were 7 to 13 years old showed the same pattern of effects as T1 (Table 2). Correlation coefficients did not differ according to Fisher *R* to *Z* (*Z* = 1.04, *p* = .15). Maternal depression symptoms also predicted child depression/anxiety in the unrelated and related groups in children younger and older than 11 years.

#### Child Gender

Associations tested separately for child gender are presented in Table 3. A significant association between maternal depression symptoms and mother-rated child depression/anxiety was found for girls in unrelated and related groups at T1. Correlations did not differ according to Fisher *R* to *Z* (*Z* = 0.31, *p* = .38). When father-rated data were used, the same pattern was evidenced for girls in unrelated and related groups and correlations did not differ according to Fisher *R* to *Z* (*Z* = 0.1.21, *p* = .11). For girls at T2 the association remained significant in unrelated and related groups for mother-rated child symptoms and correlations were not significantly different (*Z* = 0.83, *p* = .20). For boys at T1 the association was significant in the related but not in the unrelated group and this pattern was replicated with father-reported child symptoms (related: n = 210, *r* = 0.21, *p* < .001; unrelated: n = 59, *r* = 0.10, *p* = .46). However, the magnitude of associations did not differ significantly between genetically related and unrelated groups for mother (*Z* = 0.82, *p* = .38) or father (*Z* = 0.75, *p* = .23) ratings. At T2 the association for boys was significant in related and unrelated groups. Comparisons of correlations for boys and girls in the unrelated group at T1 were significant for mother (*Z* = 1.71, *p* < .05) and father (*Z* = 1.73, *p* < .05) ratings, with a larger effect observed for girls. The difference between boys and girls in the unrelated group at T2 approached significance (*Z* = 1.54, *p* = .06). Gender and age results remained the same when analyses were rerun including the previously used covariates and shared adversities (results available from first author).

#### Role of Paternal Depression Symptoms

The association between maternal depression symptoms and child depression/anxiety was tested controlling for the role of paternal depression symptoms. Associations remained significant for mother ratings of child symptoms (unrelated: n = 133, *r* = 0.31, *p* < .001; related: n = 441, *r* = 0.28, *p* < .001) and father ratings (unrelated: n = 131, *r* = 0.17, *p* < .05; related: n = 434, *r* = 0.16, *p* < .001). The association between paternal depression symptoms and child depression/anxiety was significant in the unrelated (n = 166, *r* = 0.17, *p* < .05) and related (n = 408, *r* = 0.23, *p* < .001) groups using mother-rated child symptoms at T1. Correlation coefficients did not differ according to Fisher *R* to *Z* (*Z* = 0.67, *p* = .25) and remained significant after controlling for the same covariates and shared adversities.

## Discussion

The importance of genetically sensitive designs in identifying environmental risk factors is well recognized. The aim of this study was to test the extent to which environmental links between maternal depression symptoms and child symptoms demonstrated in previous reports were explained by measurements of shared adversity and by child age and gender.

Results are consistent with an environmental link between maternal depression symptoms and child depression/anxiety. Associations were observed regardless of whether mothers and children were genetically related. There are now converging findings across different genetically sensitive designs of environmental links between maternal and offspring depression.^2,3,12^ The present findings therefore consolidate evidence that intergenerational transmission of depression symptoms is due at least in part to environmental mechanisms and does not arise entirely from shared genetic liability. The presence of an association in the unrelated group also rules out passive gene-environment correlation, which is absent in genetically unrelated mother–child pairs. The present study accounted for three measurements of shared adversity: negative life events, family income and family socioeconomic status, and depression in fathers. Adjusted associations remained significant in genetically unrelated and related parent–child pairs. This favors the suggestion that noninherited links between mother and child depression may arise from direct environmental effects and are not attributable to the extraneous variables measured in this study. This is consistent with findings that shared stressful life events and social support networks do not contribute to links between maternal and child depression.^4^ Although the covariates, family income, family social occupational class, and life events, may be genetically influenced and meaningful genetic variance may therefore be unsystematically removed, the pattern of genetic and environmental transmission was the same whether the covariates were included.

Child age is an important factor to consider because depression etiology has been found to differ developmentally, with greater genetic influences in adolescence.^18^ Significant environmental links were found when children were 4 to 10 and 7 to 13 years old. At T2 significant environmental transmission effects were found in younger children (7 to 11 years) and those in early adolescence (11 to 13 years). Significant environmental transmission effects were found in both age groups, suggesting that associations were not due to the young age of the sample and that environmental links also operate in children entering early adolescence. This concurs with previous genetically sensitive studies on transmission effects that looked at older samples.^2,3^

Tully et al.^2^ found some indication for the role of genetic factors. Although genetic influences cannot be ruled out in the present study, no clear support was found for a genetic contribution to transmission apart from in boys (see later); maternal–child associations were not stronger in the genetically related group. Interestingly, the study by Silberg et al.^3^ also reported no significant genetic influence. However, in contrast to the study by Tully et al.,^2^ our findings and those of Silberg et al.^3^ relied on symptoms rather than diagnoses of depression so it is possible that inherited transmission is more pronounced for more severe maternal depression symptoms. Indeed, a study of adolescent female twins found that the contribution of shared environmental factors differed according to the phenotype examined, with shared environmental effects observed for a broad depressive phenotype but not for major depressive disorder.^19^ Alternatively, it may be that the heritable phenotype manifests in a different way, for example, as overall levels of psychopathology in children rather than as depression symptoms per se. In contrast to these previous studies, the present study examined a composite of depression/anxiety scores. However, the pattern of maternal–child associations was found to be the same for child depression scores alone. Another consideration is that none of the designs (adoption, children of twins, IVF) are able to account for genetic heterogeneity between depression in childhood and depression in adulthood. If different genes are responsible for depression in childhood versus adulthood,^11^ then this may have the effect of decreasing the genetic transmission estimate. This is an important direction for future research.

Based on a range of studies suggesting that maternal depression may have stronger adverse effects for girls, the present study examined whether patterns of intergenerational transmission differed by gender. The significant association in genetically unrelated mother–daughter pairs indicated an environmental effect. For mother–son pairs, the association was significant only in the genetically related group, suggesting that environmental transmission was less strong for boys. These findings are in agreement with numerous nongenetic studies that have reported stronger associations between maternal depression and child depression in girls^7,8^ and suggest that for girls this effect may occur through environmental pathways because the association was of a similar magnitude in the genetically unrelated and related groups. This concurs with research suggesting that girls are more sensitive to stressful life events in general, especially those of an interpersonal nature. The significant association between maternal depression symptoms and child depression/anxiety in related but not in unrelated groups for boys does not conclusively rule out environmental transmission for boys because the difference in the magnitude of correlations was not large. Indeed, environmental transmission was seen for boys in analyses conducted at T2. Prior nongenetic studies of the association between maternal and child depression have reported stronger associations for girls only at puberty, which coincides with the emergence of the gender difference in depression prevalence.^8^ However the present study suggests that from a younger age, girls may already possess an increased vulnerability to maternal depression exposure. A recent study attests to this view.^20^

The results of this study must be interpreted in light of certain limitations. Other unmeasured shared adversities may have contributed to the intergenerational link. The association between maternal depression symptoms and child mood cannot be assumed to be unidirectional. A lack of power in the longitudinal analyses when the sample is split by conception group and gender precluded an investigation of reverse causation. Despite evidence that the association is likely to be bidirectional, research suggests that it is unlikely to be accounted for by child effects on parent symptoms alone. A recent prospective longitudinal study of parental depressive symptoms and child temperament found significant parent-to-child effects but little support for child-to-parent effects.^21^ Like other longitudinal studies, attrition affected sample retention especially for father participation. There was no evidence that this biased the results (those who dropped out of the study did not differ from those who were retained in maternal and child depression symptom scores), but it did limit the scope and power available for some T2 analyses. Significant environmental transmission but a lack of differences between the related and unrelated groups suggesting no genetic transmission could also be accounted for by the use of self-report questionnaire measurements, which are subject to greater error than interview measurements. The present study focused on symptom ratings and further studies focusing on clinical diagnoses of parent depression and on clinical outcomes in children would be of value. However, focus on symptoms is still useful to an understanding of clinical diagnoses because symptoms and disorder show similar correlates (e.g., increased rates in postpubertal girls) and similar patterns of association with risk factors (e.g., life events, family adversity).

Another issue is the use of an age-based cutoff to approximate the effects of pre- and postpuberty because this is not an exact measurement of pubertal development. However, pubertal data were not collected so this was the best available approximation. IVF may result in epigenetic modifications that lead to offspring born with imprinting disorders.^22^ In practice, however, this sample has been shown to be comparable to the normal population in terms of emotional and behavioral adjustment. This does not rule out the possibility that epigenetic modifications have occurred, although this would presumably have the effect of introducing greater phenotypic differences between parents and children in this sample compared with naturally conceived offspring and parents.

The findings have clinical implications for the design of preventive interventions: concluding that a putative causal risk factor such as parental depression symptom exposure confers its effect through an environmental mechanism suggests that this factor can be targeted to prevent and treat child and adolescent depression. In support of this, evidence suggests that changes in current maternal depression status have corresponding effects on child outcome.^23^ The environmental effect of maternal depression appears to be stronger in girls than in boys, suggesting that depression in mothers carries a particular risk effect for daughters, implying that interventions targeted at treating parental depression may have a larger impact on girls as supported by a recent meta-analysis of preventive interventions.^24^ In addition, parent depression was found to have an environmental link with anxiety/depression symptoms from childhood through to early adolescence. Little support was found for the role of genetic factors on the transmission of depression symptoms apart from for boys, and this concurs with a prior genetically sensitive study using a children-of-twins model.^3^ This does not negate the role of child- and adult-specific genetic factors in the etiology of depression symptoms, which is a well-established and consistent finding. It is likely that the etiology and transmission of depression are caused by a complex interplay between genetic and environmental factors.

## Figures and Tables

**TABLE S1:** Correlations Presented Separately for Child Depression and Anxiety Symptoms

	Genetically Related		Genetically Unrelated	
	Correlation Coefficient	n	Correlation Coefficient	n
Child depression symptoms T1				
Mother-rated child symptoms	0.25	639	0.29	207
Father-rated child symptoms	0.18	439	0.32	133
Child anxiety symptoms T1				
Mother-rated child symptoms	0.31	638	0.31	207
Father-rated child symptoms	0.23	439	0.16	133
Child depression symptoms T2	0.29	340	0.40	99
Child anxiety symptoms T2	0.27	347	0.40	100

Note: T1 = data collection in 2006; T2 = data collection in 2009.

**TABLE 1:** Testing Environmental Transmission Controlling for Shared Adversity

	Genetically Related		Genetically Unrelated	
	Partial Correlation Coefficient	n	Partial Correlation Coefficient	n
Independent negative life events				
Mother-rated child symptoms	0.30⁎⁎⁎	637	0.30⁎⁎⁎	206
Father-rated child symptoms	0.23⁎⁎⁎	434	0.26⁎⁎	131
Family income				
Mother-rated child symptoms	0.29⁎⁎⁎	614	0.33⁎⁎⁎	202
Father-rated child symptoms	0.21⁎⁎⁎	431	0.23⁎⁎	131
Family socio-occupational class				
Mother-rated child symptoms	0.31⁎⁎⁎	622	0.31⁎⁎⁎	197
Father-rated child symptoms	0.23⁎⁎⁎	431	0.25⁎⁎	131
Multiple partial correlation				
Mother-rated child symptoms	0.30⁎⁎⁎	596	0.30⁎⁎⁎	191
Father-rated child symptoms	0.22⁎⁎⁎	416	0.22⁎⁎	129

Note:

⁎⁎ *p* < .01,

⁎⁎⁎ *p* < .001.

**TABLE 2:** Associations Between Maternal Depression Symptoms and Child Depression/Anxiety Symptoms Tested Separately by Child Age

	Relatedness Group			
	Genetically Related		Genetically Unrelated	
	Correlation Coefficient	n	Correlation Coefficient	n
T1 (age 4–10 y)	0.31⁎⁎⁎	639	0.32⁎⁎⁎	207
T2 (age 7–13 y)	0.32⁎⁎⁎	352	0.44⁎⁎⁎	105
Sample split by age at T2				
>11 y old at T2	0.34⁎⁎⁎	134	0.40⁎	33
<11 y old at T2	0.32⁎⁎⁎	218	0.46⁎⁎⁎	72

Note: T1 = data collection in 2006; T2 = data collection in 2009.

⁎ *p < .05*,

⁎⁎⁎ *p < .001*.

**TABLE 3:** Associations Between Maternal Depression Symptoms and Child Depression/Anxiety Symptoms Tested Separately by Child Gender

Gender Effects	Relatedness Group			
Genetically Related		Genetically Unrelated	
Correlation Coefficient	n	Correlation Coefficient	n
Boys T1				
Mother-rated child symptoms	0.28⁎⁎⁎	329	0.18	99
Father-rated child symptoms	0.21⁎⁎⁎	210	0.10	59
Girls T1				
Mother-rated child symptoms	0.37⁎⁎⁎	309	0.40⁎⁎⁎	107
Father-rated child symptoms	0.24⁎⁎⁎	225	0.39⁎⁎⁎	107
Boys T2	0.26⁎⁎⁎	185	0.25⁎⁎	43
Girls T2	0.41⁎⁎⁎	165	0.51⁎⁎⁎	60

Note: T1 = data collection in 2006; T2 = data collection in 2009.

⁎⁎ *p < .01*,

⁎⁎⁎ *p < .001*.
